# Pneumococcal pneumonia in adults – re-emergence of vaccine serotypes: a prospective multicenter cohort study in Germany, 2020–2023

**DOI:** 10.1186/s41479-026-00204-3

**Published:** 2026-07-25

**Authors:** Christina Bahrs, Norman Rose, Grit Barten-Neiner, Carolin Fleischmann-Struzek, Mark P. G. van der Linden, Gernot Rohde, Jan Rupp, Martin Witzenrath, Jessica Rademacher, Mathias W. Pletz

**Affiliations:** 1 Division of Infectious Diseases and Tropical Medicine, Department of Medicine I, Medical University of Vienna, Vienna, Austria; 2 Present Address: Institute of Infectious Diseases and Infection Control, Jena University Hospital/Friedrich-Schiller-University, Am Klinikum 1, 07747 Jena, Germany; 3 Center for Sepsis Care and Control, Jena University Hospital/Friedrich-Schiller- University, Jena, Germany; 4 Biomedical Research in Endstage and Obstructive Lung Disease Hannover (BREATH), German Center for Lung Research (DZL), Hannover, Germany; 5 CAPNETZ STIFTUNG, Hannover, Germany; 6 Reference Laboratory for Streptococci, Department of Medical Microbiology, University Hospital (RWTH), Aachen, Germany; 7 Department of Respiratory, Intensive Care and Sleep Medicine, German Center for Lung Research (DZL), University Hospital Gießen-Marburg, Philipps University of Marburg, Marburg, Germany; 8 Infectious Disease Clinic, University Hospital Schleswig-Holstein, Campus Lübeck, Lübeck, Germany; 9 Institute of Medical Microbiology, University of Lübeck and University Hospital Schleswig-Holstein, Campus Lübeck, Lübeck, Germany; 10 Department of Infectious Diseases, Respiratory Medicine and Critical Care, Charité-Universitätsmedizin Berlin, Freie Universität Berlin and Humboldt-Universität zu Berlin, Berlin, Germany; 11 Department of Respiratory Medicine and Infectious Diseases, Hannover Medical School, Hannover, Germany; 12 Pneumologie Süderelbe, Hamburg, Germany

**Keywords:** *Streptococcus pneumoniae*, Conjugate vaccine, Community-acquired pneumonia, Serotype-specific urinary antigen detection assay, Serotype 3, SARS CoV-2 pandemic

## Abstract

The main burden of non-invasive pneumococcal diseases in adults is largely due to community-acquired pneumonia (CAP). This study aimed to investigate the distribution and dynamics of pneumococcal vaccine serotypes and to determine the proportion of CAP cases attributable to serotypes covered by 13-valent conjugate vaccine (PCV13), the 20-valent conjugate vaccine (PCV20), and the 23-valent polysaccharide vaccine (PPV23) among adults in Germany from 2020 to 2023. In this prospective multicenter cohort study, we analyzed all adult patients with CAP enrolled between January 1, 2020, and December 31, 2023, at 26 centers in Germany who provided urine samples for serotype-specific urine antigen detection (SSUAD) testing. Annual trends of pneumococcal vaccine serotypes from 2020 to 2023 were calculated for all patients and patient groups at risk using cluster-robust generalized linear models with heteroscedasticity-consistent standard errors. Of the 2,028 patients with all-cause CAP, 1,504 (1,008 patients aged ≥ 60 years, 373 younger patients with at least one comorbidity) had urine samples analyzed with SSUAD tests. Overall proportion of vaccine-type pneumococcal pneumonia among all-cause CAP for PCV13, PCV20, and PPV23 serotypes was 5.41% (95% CI 4.12–7.06%), 8.11% (95% CI 6.35–10.31%), and 8.31% (95% CI 6.27–10.93%), and serotype 3 was the most prevalent serotype (52 cases). Among patients aged ≥ 60 years, an increasing annual trend was observed for serotype 3 (OR 1.84, 95% CI 1.01–2.67), PCV13 (OR 1.89, 95% CI 0.89–2.89), PCV20 (OR 1.57, 95% CI 0.99–2.14), and PPV23 serotypes (OR 1.47, 1.04–1.91). The findings demonstrate that vaccine serotypes, particularly serotype 3, continued to circulate and reemerged among older adults with CAP in Germany.

## Key Message

“Take home” message: In Germany, S. pneumoniae has reemerged as common pathogen of pneumonia, particularly in older adults. This resurgence, observed during the ongoing SARS-CoV-2 pandemic, has been primarily driven by an increase in cases due to serotype 3.

## Introduction

The main burden of non-invasive pneumococcal diseases in adults is largely due to community-acquired pneumonia (CAP) [1, 2]. A previous CAPNETZ cohort study reported a marked shift in the etiology of community-acquired pneumonia (CAP) among adults in Germany, with SARS-CoV-2 replacing common pathogens, such as *Streptococcus pneumoniae*, after April 2020 [3]. However, a prospective, population-based study in Thuringia (ThEpiCAP), Germany, found a re-emergence of *S. pneumoniae* as a causative pathogen in adults with CAP, beginning in the second half of 2021 [4]. Therefore, this raises the question, of whether the CAPNETZ cohort, that includes patients all over Germany, provides a more robust basis for evaluating the re-emergence of *S. pneumoniae* in Germany as a predominant etiological agent and the associated implications for pneumococcal vaccine coverage. Accordingly, this prospective multicenter study aimed investigate the distribution and dynamics of pneumococcal serotypes and to determine the proportion of CAP cases attributable to serotypes covered by the 13-valent pneumococcal conjugate vaccine (PCV13), the 20-valent pneumococcal conjugate vaccine (PCV20), and the 23-valent polysaccharide vaccine (PPV23) among adults in Germany from 2020 to 2023.

## Methods

We analyzed all patients enrolled in the CAPNETZ study (DRKS00005274, Ethics No. 104/01) between January 1, 2020, and December 31, 2023, at 26 centers in Germany. Participants gave written informed consent and provided urine samples for serotype-specific urine antigen detection (SSUAD) testing [5]. Eligible subjects were adults (≥ 18 years) with radiologically confirmed CAP and at least one clinical symptom. Exclusion criteria included hospital admission within the previous 28 days or new pulmonary tuberculosis diagnosed in the past two months. In addition to conventional pneumococcal urine antigen test (*BinaxNOW*^®^ S. pneumoniae) and blood culture (BC) [1], we prospectively collected urine samples for SSUAD testing for detecting PCV13 serotypes [6] and 11 additional serotypes unique to PCV20 and PPV23 [7]. Samples were treated as previously described [8] and the analyses were performed at Pfizer’s Vaccines Research and Development Laboratory (Pearl River, NY, USA). Results were classified into “positive”, “indeterminate or missing” (both excluded from analysis) and “negative”. In accordance with STIKO’s 2016 recommendation for adult pneumococcal vaccination, individuals were classified as “at risk for pneumococcal disease” if they were aged ≥ 60 years, younger adults with a comorbidity, or severely immunosuppressed individuals [9]. We present the absolute and relative frequencies of PCV13, PCV20, and PPV23 serotypes stratified by year and patient group, along with cluster-robust confidence intervals (CIs). Annual trends from 2020 to 2023 were analyzed using cluster-robust generalized linear models with heteroscedasticity-consistent standard errors. This approach accounts for data dependencies arising from the nested data structure (e.g., patients nested within hospitals). We used the saturated model g(Y_t_) = α + β_1_T_year > 2020_ + β_2_T_year > 2021_ + β_3_T_year = 2023_, with the logit-link function g(). Due to the time coding according to a change score model using the indicator variables T_j_, the regression coefficient β_j_ represents the log. odds ratios (OR) between two consecutive years. The trend was quantified by the average OR $\:avg.OR=\frac{1}{3}{\sum\:}_{j=1}^{3}\mathrm{exp}{(\beta\:}_{j})$. Standard errors and 95% CIs of the derived parameters avg. OR and avg.∆ were calculated with delta method [10]. All statistical analyses were performed using the R software including the survey and car packages [11].

## Results

Of the 2,028 patients with all-cause CAP, 1,504 (74.16%) had urine samples analyzed with SSUAD tests. The majority were male (63.30%) and had at least one comorbidity (88.83%). Almost all received inpatient care (98.80%). The mean age was 65.5 years (range 18–99 years), with approximately one third younger than 60 years. Vaccination rates against influenza and pneumococcal diseases were 41.29% and 20.68%, respectively. The most frequent comorbidities were cardiovascular diseases (61.50%), chronic respiratory illnesses (36.17%), and malignancies (21.61%). Severe immunocompromise was identified in 8.64% of patients. Mortality was 6.25% after 28 days and increased to 11.17% by 180 days.

During the study, a total of 1,492 patients had valid SSUAD test results, of whom 131 (8.78%) patients tested positive. The SSUAD results were indeterminate in 7 patients, and data were missing for 5 patients. Among 1,482 patients tested with the BINAX NOW assay, 76 (5.13%) were positive, while 22 (1.46%) patients did not undergo the BINAX NOW test. *S. pneumoniae* was identified in BCs of 24 (2.42%) of the 990 patients who had at least one BC performed. Among patients with bacteremic pneumococcal pneumonia, the following serotypes were detected by SSUAD: serotype 3 (*n* = 8, 33.33%), serotype 8 (*n* = 3, 12.50%), serotype 4 (*n* = 1, 4.17%), serotype 10 A (*n* = 1, 4.17%), serotype 19 A (*n* = 1, 4.17%), serotype 20 (*n* = 1, 4.17%), and serotype 22 F (*n* = 1, 4.17%). In 8 (33.33%) patients with bacteremic pneumococcal CAP, no vaccine serotype could be identified by SSUAD.

Among patients with all-cause CAP, serotype 3 was the most prevalent serotype, detected by SSUAD in 52 cases including the 8 patients with *S. pneumoniae* bacteremia. This was followed by serotype 8 in 17 cases, serotype 22 F in 8 cases, serotype 6 A in 7 cases, and serotype 4 and 19 A in 6 cases each. Serotypes 11 A, 9 N, and 20 were each found in 5 cases. Overall proportion of vaccine-type pneumococcal pneumonia among all-cause CAP for PCV13, PCV20, and PPV23 serotypes was 5.41% (95% CI 4.12–7.06%), 8.11% (95% CI 6.35–10.31%), and 8.31% (95% CI 6.27–10.93%), respectively. Significantly increasing annual trends were observed for pneumococcal pneumonia detected by BINAX NOW test (OR 1.77, 95% CI 1.37–2.17), for PCV13 serotypes (OR 1.53, 95% CI 0.97–2.09), and for serotype 3 (OR 1.85, 95% 1.19–2.51). Table 1 presents the proportions of pneumococcal serotypes among all-cause CAP from 2020 to 2023, along with the serotype distributions stratified according to the STIKO classifications. Serotype 3 was the most common serotype, followed by serotype 8, in both subgroups: those aged ≥ 60 years, and those aged 18–59 years with at least one comorbidity. Notably, among patients aged ≥ 60 years, an increasing annual trend was observed for serotype 3 (OR 1.84, 95% CI 1.01–2.67), PCV13 (OR 1.89, 95% CI 0.89–2.89), PCV20 serotypes (OR 1.57, 95% CI 0.99–2.14), and PPV23 serotypes (OR 1.47, 1.04–1.91). Among 130 patients with severe immunosuppression, serotype 6 A was the most prevalent serotype, followed by serotypes 3 and 8.

**Table 1:** *Streptococcus pneumoniae* detected by conventional methods (BINAX NOW urine antigen test, blood culture) and distribution of pneumococcal vaccine serotypes detected by SSUAD in adult patients with community-acquired pneumonia and in patient subgroups per STIKO recommendation for pneumococcal vaccination (individuals ≥ 60 years, individuals 18–59 years with at-risk condition, i.e. ≥1 comorbidity, individuals ≥ 18 years with severe immunosuppression)

	Study period					Annual trends by cluster-robust logistic model OR, 95% CI ^a^
	Overall	2020	2021	2022	2023	
**≥ 18 years of age**						
Patients	*N* = 1504	*N* = 330	*N* = 375	*N* = 322	*N* = 477	
*S. pneumoniae* detected by BINAX NOW test	76/1482 (5.13%, 3.94–6.65%)	6/324 (1.85%, 0.78–4.32%)	10/373 (2.68%, 1.22–5.81%)	21/316 (6.65%, 5.28–8.34%)	39/469 (8.32%, 5.67–12.03%)	1.77, 1.37–2.17 *
*S. pneumoniae* detected by BC	24/990 (2.42%, 1.44–4.07%)	2/173 (1.16%, 0.27–4.77%)	2/225 (0.89%, 0.16–4.67%)	5/202 (2.48%, 0.90–6.66%)	15/390 (3.85%, 2.36–6.22%)	1.72, 0.42–3.03
*S. pneumoniae* detected by SSUAD	131/1492 (8.78%, 6.88–11.14%)	20/327 (6.12%, 3.13–11.61%)	24/373 (6.43%, 3.87–10.51%)	27/319 (8.46%, 5.19–13.51%)	60/473 (12.69%, 9.92–16.09%)	1.32, 0.99–1.66
PCV13 serotypes	81/1498 (5.41%, 4.12–7.06%)	9/328 (2.74%, 1.18–6.24%)	16/375 (4.27%, 2.02–8.80%)	15/319 (4.70%, 2.89–7.57%)	41/476 (8.61%, 6.01–12.21%)	1.53, 0.97–2.10 *
ST3	52/1501 (3.46%, 2.31–5.16%)	5/329 (1.52%, 0.39–5.68%)	5/375 (1.33%, 0.56–3.16%)	13/321 (4.05%, 2.69–6.05%)	29/476 (6.09%, 3.78–9.69%)	1.85, 1.19–2.51 *
Other PCV13 serotypes than ST3	28/1504 (1.86%, 1.17–2.96%)	4/330 (1.21%, 0.38–3.84%)	11/375 (2.93%, 1.17–7.12%)	2/322 (0.62%, 0.15–2.47%)	11/466 (2.31%, 1.32-4.00%)	2.15, 0.00-4.45
ST6A	7/1501 (0.47%, 0.12–1.75)	0/329 (0.00%, 0.00–0.00%)	5/375 (1.33%, 0.30–5.76%)	0/321 (0.00%, 0.00–0.00%)	2/476 (0.42%, 0.15–1.16%)	-
ST4	6/1501 (0.40%, 0.18–0.90%)	0/329 (0.00%, 0.00–0.00%)	2/375 (0.53%, 0.12–2.44%)	1/321 (0.31%, 0.04–2.23%)	3/476 (0.63%, 0.19–2.04%)	-
ST19A	6/1499 (0.40%, 0.20–0.82%)	1/329 (0.30%, 0.04–2.45%)	1/375 (0.27%, 0.06–1.18%)	1/320 (0.31%, 0.04–2.61%)	3/475 (0.63%, 0.24–1.66%)	-
ST14	2/1501 (0.13%, 0.03–0.61%)	0/329 (0.00%, 0.00–0.00%)	1/375 (0.27%, 0.03–2.28%)	0/321 (0.00%, 0.00–0.00%)	1/476 (0.21%, 0.03–1.61%)	-
ST23F	2/1500 (0.13%, 0.03–0.70%)	1/329 (0.30%, 0.05–1.85%)	1/375 (0.27%, 0.06–1.18%)	0/320 (0.00%, 0.00–0.00%)	0/476 (0.00%, 0.00–0.00%)	-
ST1	1/1501 (0.07%, 0.01–0.35%)	0/329 (0.00%, 0.00–0.00%)	0/375 (0.00%, 0.00–0.00%)	0/321 (0.00%, 0.00–0.00%)	1/476 (0.21%, 0.04–1.21%)	-
ST19F	1/1499 (0.07%, 0.01–0.59%)	0/329 (0.00%, 0.00–0.00%)	0/375 (0.00%, 0.00–0.00%)	0/319 (0.00%, 0.00–0.00%)	1/476 (0.21%, 0.02–1.95%)	-
ST5	1/1500 (0.07%, 0.01–0.59%)	1/328 (0.31%, 0.037–2.46%)	0/375 (0.00%, 0.00–0.00%)	0/321 (0.00%, 0.00–0.00%)	0/476 (0.00%, 0.00–0.00%)	-
ST18C	1/1501 (0.07%, 0.01–0.35%)	0/329 (0.00%, 0.00–0.00%)	1/375 (0.27%, 0.06–1.18%)	0/321 (0.00%, 0.00–0.00%)	0/476 (0.00%, 0.00–0.00%)	-
ST6B	1/1501 (0.07%, 0.01–0.58%)	1/329 (0.30%, 0.04–2.51%)	0/375 (0.00%, 0.00–0.00%)	0/321 (0.00%, 0.00–0.00%)	0/476 (0.00%, 0.00–0.00%)	-
ST7V, ST9V	0/1501 (0.00%, 0.00–0.00%)	0/329 (0.00%, 0.00–0.00%)	0/375 (0.00%, 0.00–0.00%)	0/321 (0.00%, 0.00–0.00%)	0/476 (0.00%, 0.00–0.00%)	-
PCV20 serotypes	121/1492 (8.11%, 6.35–10.31%)	18/327 (5.51%, 2.71–10.86%)	24/373 (6.43%, 3.87–10.51%)	24/319 (7.52%, 4.54–12.21%)	55/473 (11.63%, 8.86–15.11%)	1.33, 0.95–1.70
PCV20 unique serotypes	40/1496 (2.67%, 1.64–4.35%)	9/329 (2.74%, 0.97–7.50%)	8/373 (2.15%, 0.74–6.08%)	9/320 (2.81%, 1.46–5.35%)	14/474 (2.95%, 1.57–5.50%)	1.05, 0.62–1.48
ST8	17/1502 (1.13%, 0.63–2.03%)	4/330 (1.21%, 0.40–3.62%)	1/374 (0.27%, 0.03–2.69%)	6/322 (1.86%, 0.73–4.68%)	6/476 (1.26%, 0.58–2.71%)	2.66, 0.00-8.02
Other PCV20 serotypes than ST8	23/1504 (1.53%, 0.90–2.58%)	5/330 (1.52%, 0.55–4.09%)	7/375 (1.87%, 0.60–5.66%)	3/322 (0.93%, 0.33–2.61%)	8/477 (1.68%, 0.80–3.47%)	1.18, 0.71–1.66
ST22F	8/1501 (0.53%, 0.32–0.88%)	2/329 (0.61%, 0.18–2.08%)	2/374 (0.54%, 0.16–1.79%)	1/322 (0.31%, 0.03–2.86%)	3/476 (0.63%, 0.22–1.78%)	-
ST11A	5/1502 (0.33%, 0.15–0.77%)	1/330 (0.30%, 0.05–1.85%)	0/374 (0.00%, 0.00–0.00%)	2/322 (0.62%, 0.19–2.03%)	2/476 (0.42%, 0.10–1.70%)	-
ST15B	4/1502 (0.27%, 0.04–1.94%)	1/330 (0.30%, 0.04–2.35%)	3/374 (0.80%, 0.10–6.29%)	0/322 (0.00%, 0.00–0.00%)	0/476 (0.00%, 0.00–0.00%)	-
ST10A	3/1499 (0.20%, 0.07–0.57%)	0/330 (0.00%, 0.00–0.00%)	0/373 (0.00%, 0.00–0.00%)	0/321 (0.00%, 0.00–0.00%)	3/475 (0.63%, 0.24–1.69%)	-
ST33F	2/1502 (0.13%, 0.03–0.67%)	0/330 (0.00%, 0.00–0.00%)	2/374 (0.54%, 0.10–2.94%)	0/322 (0.00%, 0.00–0.00%)	0/476 (0.00%, 0.00–0.00%)	-
ST12F	1/1501 (0.07, 0.01–0.59%)	1/330 (0.30%, 0.04–2.38%)	0/374 (0.00%, 0.00–0.00%)	0/322 (0.00%, 0.00–0.00%)	0/475 (0.00%, 0.00–0.00%)	-
PPV23 serotypes	124/1492 (8.31%, 6.27–10.93%)	20/327 (6.12%, 3.13–11.61%)	19/373 (5.09%, 3.07–8.34%)	27/319 (8.46%, 5.19–13.51%)	58/473 (12.26%, 9.37–15.89%)	1.35, 1.03–1.67
PPV23 unique serotypes	11/1502 (0.7%, 0.73–1.16%)	2/330 (0.61%, 0.17–2.19%)	0/374 (0.00%, 0.00–0.00%)	3/322 (0.93%, 0.32–2.69%)	6/476 (1.26%, 0.60–2.64%)	-
ST9N	5/1502 (0.33%, 0.12–0.92%)	0/330 (0.00%, 0.00–0.00%)	0/374 (0.00%, 0.00–0.00%)	2/322 (0.62%, 0.19–2.03%)	3/476 (0.63%, 0.13–3.02%)	-
ST20	5/1502 (0.33%, 0.14–0.79%)	2/330 (0.61%, 0.17–2.19%)	0/374 (0.00%, 0.00–0.00%)	1/322 (0.31%, 0.03–2.99%)	2/476 (0.42%, 0.12–1.44%)	-
ST2	1/1502 (0.07%, 0.01–0.41%)	0/330 (0.00%, 0.00–0.00%)	0/374 (0.00%, 0.00–0.00%)	0/322 (0.00%, 0.00–0.00%)	1/476 (0.21%, 0.03–1.40%)	-
ST17F	0/1502 (0.00%, 0.00–0.00%)	0/330 (0.00%, 0.00–0.00%)	0/374 (0.00%, 0.00–0.00%)	0/322 (0.00%, 0.00–0.00%)	0/476 (0.00%, 0.00–0.00%)	-
**≥ 60 years of age**						
Patients	*N* = 1008	*N* = 185	*N* = 234	*N* = 247	*N* = 342	
*S. pneumoniae* detected by BINAX NOW test	47/993 (4.73%, 3.50–6.38%)	2/182 (1.10%, 0.24–4.94%)	8/233 (3.43%, 1.57–7.31%)	11/242 (4.55%, 3.25–6.32%)	26/336 (7.74%, 4.95–11.90%)	2.11, 0.44–3.77 *
*S. pneumoniae* detected by BC	20/679 (2.95%, 1.71–5.04%)	2/99 (2.02%, 0.45–8.56%)	1/147 (0.68%, 0.06–7.87%)	5/152 (3.29%, 1.31–8.03%)	12/281 (4.27%, 2.42–7.44%)	2.20, 0.00-5.93
*S. pneumoniae* detected by SSUAD	82/1000 (8.20%, 6.22–10.73%)	11/185 (5.95%, 3.46–10.03%)	15/232 (6.47%, 4.12–10.01%)	13/245 (5.31%, 2.84–9.69%)	43/338 (12.72%, 9.59–16.69%)	1.50, 1.02–1.98 *
PCV13 serotypes	52/1005 (5.17%, 3.68–7.23%)	4/185 (2.16%, 0.77–5.94%)	11/234 (4.70%, 2.58–8.42%)	8/245 (3.27%, 1.73–6.07%)	29/341 (8.50%, 5.86–12.20%)	1.89, 0.89–2.89 *
ST3	34/1006 (3.38%, 1.96–5.76%)	2/185 (1.08%, 0.25–4.60%)	4/234 (1.71%, 0.61–4.73%)	7/246 (2.85%, 1.46–5.49%)	21/341 (6.16%, 3.56–10.46%)	1.84, 1.01–2.67 *
Other PCV13 serotypes than ST3	17/1008 (1.69%, 1.14–2.49%)	2/185 (1.08%, 0.24–4.83%)	7/234 (2.99%, 1.29–6.80%)	1/247 (0.41%, 0.06–2.82%)	7/342 (2.05%, 1.16–3.60%)	2.70, 0.00-6.53
ST4	4/1006 (0.40%, 0.19–0.81%)	0/185 (0.00%, 0.00–0.00%)	1/234 (0.43%, 0.04–4.38%)	1/246 (0.41%, 0.06–2.83%)	2/341 (0.59%, 0.20–1.72%)	-
ST6A	4/1006 (0.40%, 0.08–1.95%)	0/185 (0.00%, 0.00–0.00%)	3/234 (1.28%, 0.33–4.90%)	0/246 (0.00%, 0.00–0.00%)	1/341 (0.29%, 0.05–1.59%)	-
ST19A	4/1005 (0.40%, 0.20–0.79%)	1/185 (0.54%, 0.06–4.47%)	1/234 (0.43%, 0.11–1.66%)	0/246 (0.00%, 0.00–0.00%)	2/340 (0.59%, 0.14–2.43%)	-
ST14	2/1006 (0.20%, 0.04–0.92%)	0/185 (0.00%, 0.00–0.00%)	1/234 (0.43%, 0.05–3.67%)	0/246 (0.00%, 0.00–0.00%)	1/341 (0.29%, 0.04–2.22%)	-
ST19F	1/1005 (0.10%, 0.01–0.90%)	0/185 (0.00%, 0.00–0.00%)	0/234 (0.00%, 0.00–0.00%)	0/245 (0.00%, 0.00–0.00%)	1/341 (0.29%, 0.03–2.68%)	-
ST18C	1/1006 (0.10%, 0.020–0.49%)	0/185 (0.00%, 0.00–0.00%)	1/234 (0.43%, 0.11–1.66%)	0/246 (0.00%, 0.00–0.00%)	0/341 (0.00%, 0.00–0.00%)	-
ST6B	1/1006 (0.10%, 0.01–0.89%)	1/185 (0.54%, 0.06–4.63%)	0/234 (0.00%, 0.00–0.00%)	0/246 (0.00%, 0.00–0.00%)	0/341 (0.00%, 0.00–0.00%)	-
ST1, ST5, ST7V, ST9V, ST23F	0/1006 (0.00%, 0.00–0.00%)	0/185 (0.00%, 0.00–0.00%)	0/234 (0.00%, 0.00–0.00%)	0/246 (0.00%, 0.00–0.00%)	0/341 (0.00%, 0.00–0.00%)	-
PCV20 serotypes	76/1000 (7.60%, 5.77–9.96%)	9/185 (4.87%, 2.84–8.22%)	15/232 (6.47%, 4.12–10.01%)	12/245 (4.90%, 2.62–8.98%)	40/338 (11.83%, 8.54–16.18%)	1.57, 0.99–2.14 *
PCV20 unique serotypes	24/1001 (2.40%, 1.39–4.12%)	5/185 (2.70%, 1.01–7.01%)	4/232 (1.72%, 0.41–7.02%)	4/245 (1.63%, 0.68–3.87%)	11/339 (3.25%, 1.50–6.90%)	1.20, 0.60–1.80
ST8	9/1006 (0.90%, 0.49–1.62%)	2/185 (1.08%, 0.32–3.59%)	1/233 (0.43%, 0.04–4.39%)	3/247 (1.22%, 0.47–3.10%)	3/341 (0.88%, 0.26–2.95%)	-
Other PCV20 serotypes than ST8	15/1008 (1.49%, 0.75–2.92%)	3/185 (1.62%, 0.62–4.16%)	3/234 (1.28%, 0.24–6.45%)	1/247 (0.41%, 0.04–3.82%)	8/342 (2.34%, 1.05–5.12%)	2.33, 0.00-5.89
ST22F	5/1006 (0.50%, 0.21–1.20%)	0/185 (0.00%, 0.00–0.00%)	1/233 (0.43%, 0.04–4.32%)	1/247 (0.41%, 0.04–3.82%)	3/341 (0.88%, 0.28–2.75%)	-
ST11A	3/1006 (0.30%, 0.12–0.74%)	1/185 (0.54%, 0.09–3.06%)	0/233 (0.00%, 0.00–0.00%)	0/247 (0.00%, 0.00–0.00%)	2/341 (0.59%, 0.15–2.30%)	-
ST10A	3/1003 (0.30%, 0.10–0.92%)	0/185 (0.00%, 0.00–0.00%)	0/232 (0.00%, 0.00–0.00%)	0/246 (0.00%, 0.00–0.00%)	3/340 (0.88%, 0.31–2.47%)	-
ST15B	3/1006 (0.30%, 0.04–2.22%)	1/185 (0.54%, 0.07–4.37%)	2/233 (0.86%, 0.10–6.75%)	0/247 (0.00%, 0.00–0.00%)	0/341 (0.00%, 0.00–0.00%)	-
ST12F	1/1005 (0.10%, 0.01–0.92%)	1/185 (0.54%, 0.06–4.47%)	0/233 (0.00%, 0.00–0.00%)	0/247 (0.00%, 0.00–0.00%)	0/340 (0.00%, 0.00–0.00%)	-
ST33F	0/1006 (0.00%, 0.00–0.00%)	0/185 (0.00%, 0.00–0.00%)	0/233 (0.00%, 0.00–0.00%)	0/247 (0.00%, 0.00–0.00%)	0/341 (0.00%, 0.00–0.00%)	-
PPV23 serotypes	78/1000 (7.80%, 5.62–10.73%)	11/185 (5.95%, 3.46–10.03%)	12/232 (5.17%, 2.74–9.55%)	13/245 (5.31%, 2.84–9.69%)	42/338 (12.43%, 8.99–16.94%)	1.47, 1.04–1.91 *
PPV23 unique serotypes	6/1006 (0.60%, 0.31–1.13%)	2/185 (1.08%, 0.31–3.70%)	0/233 (0.00%, 0.00–0.00%)	1/247 (0.41%, 0.05–3.54%)	3/341 (0.88%, 0.36–2.14%)	-
ST20	4/1006 (0.40%, 0.16–0.99%)	2/185 (1.08%, 0.31–3.70%)	0/233 (0.00%, 0.00–0.00%)	0/247 (0.00%, 0.00–0.00%)	2/341 (0.59%, 0.18–1.90%)	-
ST9N	2/1006 (0.20%, 0.05–0.88%)	0/185 (0.00%, 0.00–0.00%)	0/233 (0.00%, 0.00–0.00%)	1/247 (0.41%, 0.05–3.54%)	1/341 (0.29%, 0.04–2.31%)	-
ST2, ST17F	0/1006 (0.00%, 0.00–0.00%)	0/185 (0.00%, 0.00–0.00%)	0/233 (0.00%, 0.00–0.00%)	0/247 (0.00%, 0.00–0.00%)	0/341 (0.00%, 0.00–0.00%)	-
**18–59 years with at-risk condition**						
Patients	*N* = 373	*N* = 97	*N* = 105	*N* = 64	*N* = 107	
*S. pneumoniae* detected by BINAX NOW test	21/369 (5.69%, 3.82–8.41%)	3/96 (3.13%, 0.91–10.16%)	1/105 (0.95%, 0.17–5.22%)	6/63 (9.52%, 4.51–19.01%)	11/105 (10.48%, 4.70-21.73%)	4.12, 0.00-11.23
*S. pneumoniae* detected by BC	4/240 (1.67%, 0.60–4.52%)	0/48 (0.00%, 0.00–0.00%)	1/58 (1.72%, 0.30–9.29%)	0/43 (0.00%, 0.00–0.00%)	3/91 (3.30%, 0.99–10.39%)	-
*S. pneumoniae* detected by SSUAD	39/369 (10.57%, 7.66–14.41%)	7/94 (7.45%, 2.79–18.42%)	8/105 (7.62%, 3.05–17.78%)	11/63 (17.46%, 11.65–25.34%)	13/107 (12.15%, 7.77–18.51%)	1.42, 0.67–2.16
PCV13 serotypes	25/370 (6.76%, 4.82–9.40%)	4/95 (4.21%, 1.18–13.93%)	5/105 (4.76%, 1.40-14.98%)	6/63 (9.52%, 4.78–18.09%)	10/107 (9.35%, 5.45–15.57%)	1.41, 0.65–2.16
ST3	16/372 (4.30%, 2.90–6.34%)	2/96 (2.08%, 0.23–16.23%)	1/105 (0.95%, 0.17–5.22%)	6/64 (9.38%, 4.67–17.93%)	7/107 (6.54%, 3.18–12.98%)	3.96, 0.00-10.52
Other PCV13 serotypes than ST3	9/373 (2.41%, 1.00-5.69%)	2/97 (2.06%, 0.50–8.15%)	4/105 (3.81%, 1.27–10.89%)	0/64 (0.00%, 0.00–0.00%)	3/107 (2.80%, 1.36–5.71%)	-
ST6A	3/372 (0.81%, 0.28–2.30%)	0/96 (0.00%, 0.00–0.00%)	2/105 (1.91%, 0.33–10.15%)	0/64 (0.00%, 0.00–0.00%)	1/107 (0.94%, 0.17–4.88%)	-
ST23F	2/371 (0.54%, 0.08–3.39%)	1/96 (1.04%, 0.14–7.18%)	1/105 (0.95%, 0.17–5.22%)	0/63 (0.00%, 0.00–0.00%)	0/107 (0.00%, 0.00–0.00%)	-
ST4	1/372 (0.27, 0.06–1.29%)	0/96 (0.00%, 0.00–0.00%)	1/105 (0.95%, 0.16–5.35%)	0/64 (0.00%, 0.00–0.00%)	0/107 (0.00%, 0.00–0.00%)	-
ST19A	1/371 (0.27%, 0.03–2.34%)	0/96 (0.00%, 0.00–0.00%)	0/105 (0.00%, 0.00–0.00%)	0/63 (0.00%, 0.00–0.00%)	1/107 (0.94%, 0.10–7.87%)	-
ST1	1/372 (0.27%, 0.04–1.71%)	0/96 (0.00%, 0.00–0.00%)	0/105 (0.00%, 0.00–0.00%)	0/64 (0.00%, 0.00–0.00%)	1/107 (0.94%, 0.13–6.52%)	-
ST5	1/371 (0.27%, 0.03–2.49%)	1/95 (1.05%, 0.12–8.83%)	0/105 (0.00%, 0.00–0.00%)	0/64 (0.00%, 0.00–0.00%)	0/107 (0.00%, 0.00–0.00%)	-
ST19F	0/371 (0.00%, 0.00–0.00%)	0/96 (0.00%, 0.00–0.00%)	0/105 (0.00%, 0.00–0.00%)	0/63 (0.00%, 0.00–0.00%)	0/107 (0.00%, 0.00–0.00%)	-
ST14, ST18C, ST6B, ST7V, ST9V	0/372 (0.00%, 0.00–0.00%)	0/96 (0.00%, 0.00–0.00%)	0/105 (0.00%, 0.00–0.00%)	0/64 (0.00%, 0.00–0.00%)	0/107 (0.00%, 0.00–0.00%)	-
PCV20 serotypes	37/369 (10.03%, 6.95–14.26%)	7/94 (7.45%, 2.79–18.42%)	8/105 (7.62%, 3.05–17.78%)	10/63 (15.87%, 9.40-25.56%)	12/107 (11.22%, 6.80-17.95%)	1.33, 0.70–1.95
PCV20 unique serotypes	12/372 (3.22%, 1.53–6.70%)	3/96 (3.13%, 0.71–12.69%)	3/105 (2.86%, 0.95–8.27%)	4/64 (6.25%, 2.78–13.48%)	2/107 (1.87%, 0.47–7.10)	1.16, 0.25–2.06
ST8	5/373 (1.34%, 0.51–3.51%)	1/97 (1.03%, 0.13–7.96%)	0/105 (0.00%, 0.00–0.00%)	2/64 (3.13%, 0.58–15.09%)	2/107 (1.87%, 0.47–7.10%)	-
Other PCV20 serotypes than ST8	7/373 (1.88%, 0.94–3.70%)	2/97 (2.06%, 0.53–7.63%)	3/105 (2.86%, 0.95–8.27%)	2/64 (3.13%, 1.15–8.21%)	0/107 (0.00%, 0.00–0.00%)	-
ST22F	3/372 (0.81%, 0.22–2.96%)	2/96 (2.08%, 0.54–7.68%)	1/105 (0.95%, 0.17–5.22%)	0/64 (0.00%, 0.00–0.00%)	0/107 (0.00%, 0.00–0.00%)	-
ST11A	2/373 (0.54%, 0.17–1.70%)	0/97 (0.00%, 0.00–0.00%)	0/105 (0.00%, 0.00–0.00%)	2/64 (3.13%, 1.15–8.21%)	0/107 (0.00%, 0.00–0.00%)	-
ST15B, ST33F	1/373 (0.27%, 0.03–2.11%)	0/97 (0.00%, 0.00–0.00%)	1/105 (0.95%, 0.10–8.15%)	0/64 (0.00%, 0.00–0.00%)	0/107 (0.00%, 0.00–0.00%)	-
ST10A, ST12F	0/373 (0.00%, 0.00–0.00%)	0/97 (0.00%, 0.00–0.00%)	0/105 (0.00%, 0.00–0.00%)	0/64 (0.00%, 0.00–0.00%)	0/107 (0.00%, 0.00–0.00%)	-
PPV23 serotypes	36/369 (9.76%, 7.16–13.17%)	7/94 (7.45%, 2.79–18.42%)	6/105 (5.71%, 2.85–11.14%)	11/63 (17.46%, 11.65–25.34%)	12/107 (11.22%, 6.84–17.85%)	1.61, 0.74–2.49
PPV23 unique serotypes	3/373 (0.80%, 0.26–2.43%)	0/97 (0.00%, 0.00–0.00%)	0/105 (0.00%, 0.00–0.00%)	1/64 (1.56%, 0.43–5.56%)	2/107 (1.87%, 0.54–6.31%)	-
ST9N	2/373 (0.54%, 0.17–1.72%)	0/97 (0.00%, 0.00–0.00%)	0/105 (0.00%, 0.00–0.00%)	1/64 (1.56%, 0.43–5.56%)	1/107 (0.94%, 0.09–8.67%)	-
ST2	1/373 (0.27%, 0.06–1.29%)	0/97 (0.00%, 0.00–0.00%)	0/105 (0.00%, 0.00–0.00%)	0/64 (0.00%, 0.00–0.00%)	1/107 (0.94%, 0.17–4.88%)	-
ST20, ST17F	0/373 (0.00%, 0.00–0.00%)	0/97 (0.00%, 0.00–0.00%)	0/105 (0.00%, 0.00–0.00%)	0/64 (0.00%, 0.00–0.00%)	0/107 (0.00%, 0.00–0.00%)	-
**≥ 18 years of age with severe immunosuppression**						
Patients	*N* = 130	*N* = 11	*N* = 34	*N* = 32	*N* = 53	
*S. pneumoniae* detected by BINAX NOW test	9/129 (6.98%, 3.01–15.33%)	0/11 (0.00%, 0.00–0.00%)	0/34 (0.00%, 0.00–0.00%)	2/31 (6.45%, 1.01–31.76%)	7/53 (13.21%, 4.17–34.74%)	-
*S. pneumoniae* detected by BC	2/107 (1.87%, 0.55–6.11%)	0/8 (0.00%, 0.00–0.00%)	0/25 (0.00%, 0.00–0.00%)	1/27 (3.70%, 0.72–17.05%)	1/47 (2.13%, 0.36–11.47%)	-
*S. pneumoniae* detected by SSUAD	8/130 (6.15%, 3.20-11.51%)	0/11 (0.00%, 0.00–0.00%)	2/34 (5.88%, 2.96–11.36%)	2/32 (6.25%, 1.33–24.78%)	4/53 (7.55%, 2.64–19.71%)	-
PCV13 serotypes	6/130 (4.62%, 2.26–9.18%)	0/11 (0.00%, 0.00–0.00%)	2/34 (5.88%, 2.96–11.36%)	0/32 (0.00%, 0.00–0.00%)	4/53 (7.55%, 2.64–19.71%)	-
ST6A	3/130 (2.31%, 0.62–8.26%)	0/11 (0.00%, 0.00–0.00%)	2/34 (5.88%, 2.96–11.36%)	0/32 (0.00%, 0.00–0.00%)	1/53 (1.89%, 0.31–10.56%)	-
ST3	2/130 (1.54%, 0.63–3.73%)	0/11 (0.00%, 0.00–0.00%)	0/34 (0.00%, 0.00–0.00%)	0/32 (0.00%, 0.00–0.00%)	2/53 (3.77%, 1.15–11.64%)	-
ST19F	1/130 (0.77%, 0.06–8.55%)	0/11 (0.00%, 0.00–0.00%)	0/34 (0.00%, 0.00–0.00%)	0/32 (0.00%, 0.00–0.00%)	1/53 (1.89%, 0.14–20.48%)	-
ST1, ST4, ST5, ST6B, ST7V, ST9V, ST14, ST18C, ST19A, ST23F	0/130 (0.00%, 0.00–0.00%)	0/11 (0.00%, 0.00–0.00%)	0/34 (0.00%, 0.00–0.00%)	0/32 (0.00%, 0.00–0.00%)	0/53 (0.00%, 0.00–0.00%)	-
PCV20 serotypes	8/130 (6.15%, 3.20-11.51%)	0/11 (0.00%, 0.00–0.00%)	2/34 (5.88%, 2.96–11.36%)	2/32 (6.25%, 1.33–24.78%)	4/53 (7.55%, 2.64–19.71%)	-
PCV20 unique serotypes	2/130 (1.54%, 0.47–4.95%)	0/11 (0.00%, 0.00–0.00%)	0/34 (0.00%, 0.00–0.00%)	2/32 (6.25%, 1.33–24.78%)	0/53 (0.00%, 0.00–0.00%)	-
ST8	2/130 (1.54%, 0.47–4.95%)	0/11 (0.00%, 0.00–0.00%)	0/34 (0.00%, 0.00–0.00%)	2/32 (6.25%, 1.33–24.78%)	0/53 (0.00%, 0.00–0.00%)	-
Other PCV20 serotypes than ST8	0/130 (0.00%, 0.00–0.00%)	0/11 (0.00%, 0.00–0.00%)	0/34 (0.00%, 0.00–0.00%)	0/32 (0.00%, 0.00–0.00%)	0/53 (0.00%, 0.00–0.00%)	-
PPV23 serotypes	5/130 (3.85%, 1.85–7.83%)	0/11 (0.00%, 0.00–0.00%)	0/34 (0.00%, 0.00–0.00%)	2/32 (6.25%, 1.33–24.78%)	3/53 (5.66%, 1.81–16.33%)	-
PPV23 unique serotypes	0/130 (0.00%, 0.00–0.00%)	0/11 (0.00%, 0.00–0.00%)	0/34 (0.00%, 0.00–0.00%)	0/32 (0.00%, 0.00–0.00%)	0/53 (0.00%, 0.00–0.00%)	-

Data are presented as No./total No. (relative frequencies in %, 95% confidence intervals)

Abbreviations: BC – blood culture, SSUAD – serotype specific urinary antigen detection assay, PCV13–13 valent pneumococcal conjugate vaccine; PCV20–20-valent pneumococcal conjugate vaccine; PPV23–23-valent pneumococcal polysaccharide vaccine, ST – serotype, STIKO – German Standing Committee on Immunization, at-risk condition – one or more chronic comorbidities predisposing to pneumococcal disease as defined by STIKO

aAnnual trends in detecting serotypes between 2020 and 2023 were estimated and tested using cluster-robust generalized linear models with heteroscedasticity consistent standard errors. Significant p-values < 0.05 are marked with an asterix

## Discussion

The findings demonstrate that vaccine serotypes of *S. pneumoniae*, particularly serotype 3, continued to circulate during the ongoing SARS-CoV-2 pandemic and reemerged among older adults with CAP in Germany, potentially reflecting waning immunity or incomplete vaccine effectiveness [12, 13]. Similarly, a recent prospective observational study on *S. pneumoniae* carriage among adults in Portugal revealed the continued presence of serotype 3 and other non-PCV13 vaccine serotypes covered by broader PCVs among civil servants aged 18 years and older during the SARS-CoV-2 pandemic [14]. A recent review on the global distribution of pneumococcal diseases in adults, encompassing both invasive pneumococcal disease and pneumococcal pneumonia, indicated that diseases caused by serotype 3 persist in both high-income and non-high-income countries. However, the prevalence of residual and emerging serotypes varies by region: serotype 8 is more common in Europe and Asia, while serotype 4 is more prevalent in the United States and Canada [15]. As pneumococcal vaccination offer and schedules in adults vary widely among European countries [16], this prospective analysis should be interpreted in light of the national vaccination coverage in Germany (https://www.rki.de/vacmap): During the study period, pneumococcal vaccination rates (≥ 1 dose) were 11.3–13.2% among 60–64-year-olds and 38.9–40.2% among 70–74-year-olds. National surveillance does not distinguish between conjugate vaccines and PPV23; however, PPV23 remained the standard adult vaccine until 2023 and likely accounts for the majority of vaccinations.

An important limitation of the study is that the SSUAD utilized does not cover 8 additional serotypes (15 A, 15 C, 16 F, 23 A, 23B, 24 F, 31, 35B) included in the new 21-valent PCV Capvaxive (V116) [17]. However, a recent multicenter study conducted between August 2022 and September 2024, involved 514 adult patients with CAP across seven hospitals located in six of the 16 states in Germany. This study assessed the distribution of 30 pneumococcal serotypes included in PCV15 and V116 using SSUAD assays. Among adults aged 60 years and older hospitalized with CAP, the analysis of Karssen et al. indicated that 13.5% of patients had at least one detected pneumococcal serotype contained in V116, compared to 12.6% for PCV20 serotypes, excluding serotype 15B, which was not included in the study-specific SSUAD assays [18].

In conclusion, *S. pneumoniae* has reemerged as a common CAP-pathogen, particularly among individuals aged ≥ 60 years in the CAPNETZ patient cohort. This resurgence during the SARS-CoV-2 pandemic has been primarily driven by an increase in cases of serotype 3 (1.08% of all-cause CAP in 2020 versus 6.16% of all-cause CAP in 2023). In 2023, PCV20 still showed a high coverage of all-cause CAP in adults (11.83% for age group ≥60 years and 11.22% for younger patients with comorbidities), reaching nearly prepandemic levels [8, 19]. Our data show (i) an increase of serotype 3, PCV13, PCV20 and PPV23 serotypes among all-cause CAP cases in older adults between 2020 and 2023 and (ii) that the gap in the coverage between PCV20 and PPV23 remains small.

## Data Availability

Data is provided within the manuscript.

## Abbreviation

BC: Blood culture
CAP: Community-acquired pneumonia
CI: Confidence interval
OR: Odds ratio
PCV13: 13-valent conjugate vaccine
PCV20: 20-valent conjugate vaccine
PPV23: 23-valent pneumococcal polysaccharide vaccine
STIKO: German Standing Committee on Immunization
SSUAD: Serotype-specific multiplex urinary antigen detection assay
